# Demographics and management of outpatient concussion visits among neurologists and non-neurologists: 2006–2016

**DOI:** 10.2217/cnc-2020-0008

**Published:** 2020-08-04

**Authors:** Patrick D Asselin, Rebekah Mannix

**Affiliations:** 1 Division of General Pediatrics, Boston Children’s Hospital, 300 Longwood Avenue, Boston, MA 02115, USA; 2 Division of Emergency Medicine; Boston Children’s Hospital; Boston, MA 02115, USA

**Keywords:** concussion, epidemiology, management, mTBI, neurology

## Abstract

**Aim:**

Describe the patient demographics and management of outpatient concussion visits, focusing on neurologists.

**Materials & methods:**

We used the National Ambulatory Medical Care Survey to provide national estimates on the demographics and clinical decisions of concussion visits from 2006 to 2016, which were identified with International Classification of Disease-9/10 codes.

**Results:**

From 2006 to 2016, there were an estimated 11 million visits nationally. Neurologists saw significantly more patients over 18 years old and more nonacute care compared with non-neurologists. Neurologists performed imaging and prescribed new medications at similar rates as non-neurologists. Non-neurology subspecialties had a significant increase in visits during the study period.

**Conclusion:**

Neurologists saw older patients and more subacute patient care with similar rates prescribing new medications and imaging. Non-neurology subspecialists are more involved in concussions than previously.

## Lay abstract

The goal of this study was to describe differences in patient characteristics and treatment patterns based on outpatient provider type, with an emphasis on comparing neurologists to other providers. We used a national survey database to provide national estimates on the patient characteristics and treatment decisions of concussion visits from 2006 to 2016, which were identified using diagnosis codes. We estimated 11 million outpatient concussion visits during the study period, 9% of which were seen by neurologists. We found neurologists took care of older patients and patients later in the course of their concussion compared with non-neurologists. Neurologists performed imaging and prescribed new medications at similar rates as non-neurologists. Non-neurology subspecialties have become more involved in concussion care during our study period.

Concussions are a common injury with 800,000–1 million outpatient visits per year in the USA [1,2]. Increased public awareness of concussions, potential long-term effects and proposed mechanisms of injury has led to further interest in understanding the pathophysiology of concussions and new methods of diagnosis [3]. Concussions are defined as bodily impacts that lead to a functional brain injury with often short-lived neurologic dysfunction with sequential recovery not explained by other factors [4]. The management of concussions is changing with the increasing understanding of concussion’s pathophysiology.

The acute management of concussions has had some consensus with protocolized return to activity and recommendations from the American Academy of Neurology (AAN; MN, USA) [5,6]. Most of these recommendations have focused on sports-related concussion (SRC) [6]. Despite this focus, there are no data regarding neurologists’ involvement in concussion care. The acute management is often handled by front-line providers, such as primary care physicians (PCP), with an increasing number of visits over the past several years among all providers [7,8]. Management of protracted concussion symptoms in terms of medications and rehabilitation among concussion specialists has been poorly studied [5]. Several studies have described the epidemiology of outpatient concussion visits among all providers [1,7,9,10]. The demographics and other clinical decisions of specialty providers, specifically neurologists, have not been well described in the ambulatory setting. In addition, there is a lack of data comparing neurologists’ demographics and decision making with other concussion providers. We propose to describe the demographics and management of concussed patients treated by neurologists and how they compare with other providers.

## Materials & methods

This study is a cross-sectional study of data collected in the National Ambulatory Medical Care Survey (NAMCS) from 2006 to 2016. The NAMCS is an annual, national probability sample of office visits made to nonfederal, office-based physicians and community health centers in the USA performed by the Centers for Disease Control and Prevention (GA, USA) at the National Center for Health Statistics (MD, USA). Physicians are randomly selected and provide data on approximately 30 patient visits. Visit information is collected during a randomly assigned 1-week reporting period each year by trained staff members at the offices with monitoring by NAMCS field representatives. NAMCS data collection is a multistage probability design to minimize bias while reporting national estimates; however, with small visit numbers (<30) such bias is possible. All NAMCS datasets are publicly available.

### Study population

We identified visits of patients of any age who presented to an office-based provider with a diagnosis of concussion. These patients were identified based on International Classification of Disease, Ninth Revision, Clinical Medicine (ICD-9-CM) and International Classification of Disease, Tenth Revision, Clinical Medicine (ICD-10-CM) codes corresponding to 850.0–850.9 (includes 850.11 & 850.12), 959.01, S06.0X0A, S06.0X1A, S06.0X1D, S06.0X9A, S06.0X0D, S09.90XA, S09.90XD [1,7,10–12].

### Study protocol

The following variables were examined to characterize patients and the outpatient visit: patient demographics (e.g., age, sex, ethnicity, race), type of insurance (e.g., private and not private), month and year of each visit, acuity of problem, diagnosis, imaging ordered, medications prescribed, doctor’s specialty, regional location and whether the patient was referred for the concussion visit. An acute problem was defined as less than or equal to 30 days from injury. The institutional review board at Boston Children’s Hospital (MA, USA) approved this study and deemed it exempt.

### Outcome measures

The primary outcome was to compare the demographics of patients seen in the ambulatory neurology setting with all other concussion providers, such as PCP or sports medicine. Secondary outcomes were clinical decisions made by concussion providers (medications prescribed, new medications prescribed, imaging ordered or procedures for concussed individuals) and temporal trends in outpatient visits. To perform the comparisons between the specialty groups, we grouped the 15 NAMCS specialty categories into either two groups – neurology and non-neurology – or three groups – neurology, primary care and non-neurology subspecialty. In the NAMCS specialty categories, there is only a category of neurologist with no pediatric neurologist sub grouping. For the two-group comparison, the neurology group only included the physicians with neurology as their specialty designation, while the non-neurology group was all other physicians. For the three-group comparison, the neurology group only included physicians designated as neurologists; the primary care group included general medicine/family practice, internal medicine and pediatricians and the non-neurology subspecialty group included all other physicians. For further specific specialty category designations, NAMCS provides yearly documentation of the survey specifying the different specialty categories.

### Data analysis

The survey data were analyzed using the sampled visit weight that is the product of the corresponding sampling fractions at each stage in the sample design. The sampling weights have been adjusted by the National Center for Health Statistics for survey nonresponse within time of year, geographic region, urban/rural and ownership designations, yielding an unbiased national estimate of ambulatory visit occurrences, percentages and characteristics. The weights, strata and primary sampling unit design variables provided by the NAMCS were used for all analyses. We used descriptive statistics for the primary outcome, with appropriate weighting, to account for the survey sampling methodology. We limited national estimates to those where visit numbers were >30 to avoid bias as previously described. For the specialty comparisons, we used survey weighted χ^2^ testing to assess differences between outcome measures between specialty categories. All analyses were performed using the statistical program, R [13–16]. Unless otherwise noted, percentages are expressed as survey-weighted proportions and all p-values are two-sided. Statistical significance has been defined as an adjusted p-value of less than 0.05. p-values are adjusted using Bonferroni correction.

## Results

Demographics and clinical decision-making categories are shown in Tables 1 and 2 with associated national estimates. During the study period, there were 447 visits, which represented 11,725,390 concussion visits. Approximately 53% of visits were pediatric patients (≤18 years old) during the study period (95% CI: 43.8–61.4%), which represented 6,165,802 visits. Of all visits, 9% of visits (95% CI: 5.8–13.1%) were seen by neurologists during the study period, representing 1,106,477 visits.

**Table 1.** Demographics of all patients with diagnosis of concussion in National Ambulatory Medical Care Survey database.

	Raw n	National estimates, n (%)
**Age, years**		
≤18	220	6,165,802 (53)
>18	247	5,559,588 (47)
**Sex**		
Female	208	5,144,093 (44)
Male	259	6,581,297 (56)
**Race**		
White	404	10,336,491 (88)
Black	43	808,954 (7)
Other race	20^†^	
**Ethnicity**		
Hispanic	71	1,857,404 (16)
Nonhispanic	396	9,867,986 (84)
**Insurance type**		
Private	245	6,375,961 (54)
Public	116	3,029,212 (26)
All others	106	2,320,217 (20)
**Geographic location**		
Northeast	111	3,484,196 (30)
Midwest	119	2,569,897 (22)
South	115	2,628,658 (22)
West	122	3,042,640 (26)
**Metropolitan**	425	10,767,505 (92)
Nonmetropolitan	42	957,885 (8)
**Practice type**		
Primary care	277	7,702,959 (66)
Neurology	97	1,106,477 (9)
Other	93	2,915,953 (25)
**Type of visit**		
Acute	333	9,029,508 (77)
Nonacute	134	2,695,882 (23)

† Signifies raw visits <30 and national estimates may be unreliable.

**Table 2.** Clinical decisions for all patient encounters.

Clinical decisions	Raw n	National estimates, n (%)
Imaging	78	2,012,906 (17)
No imaging	389	9,712,483 (83)
Referred	106	1,874,319 (16)
Not referred	361	9,851,071 (84)
Medications	262	6,580,846 (56)
No medications	205	5,144,544 (44)

When comparing the visit characteristics between neurologists and other concussion providers, we found that neurologists saw a larger proportion of patients over the age of 18 (χ^2^ = 15.1; p = 0.0088, Table 3). In addition, we found neurologists had a larger proportion of their visit types designated as nonacute (χ^2^ = 39.23; p < 0.001, Table 3). The remaining demographic variables did not show significant differences between neurologists and non-neurologists.

**Table 3.** Demographic comparison between neurology encounters and other providers.

	Neurology only		Non-neurology		χ2	p-value
	Raw n	National estimates, n (%)	Raw n	National estimates, n (%)		
**Age, years**					15.09	0.0088
≤18	20^†^		200	5,891,584 (55)		
>18	77	832,259 (75)	170	4,727,329 (45)		
**Sex**					0.21	1
Female	46	521,101 (47)	162	4,622,993 (44)		
Male	51	585,377 (53)	208	5,995,920 (56)		
**Race**					6.82	1
White	83	876,988 (79)	321	9,459,503 (89)		
Black	8^†^		35	722,969 (7)		
Other	6^†^		14^†^			
**Ethnicity**					0.36	1
Hispanic	17^†^	140,652 (13)	54	1,716,751 (16)		
Nonhispanic	80	965,825 (87)	316	8,902,161 (84)		
**Insurance type**					13.47	0.27
Private	51	586,788 (53)	194	5,789,173 (55)		
Public	22^†^		94	2,727,098 (26)		
Other	24^†^		82	2,102,641 (20)		
**Region**					2.1	1
Northeast	23^†^		88	3,211,662 (30)		
Midwest	15^†^		104	2,375,001 (22)		
South	25^†^		90	2,366,329 (22)		
West	34	376,718 (34)	88	2,665,921 (25)		
**Metropolitan**	95	1,082,532 (98)	330	9,684,972 (91)	2.34	0.33
Nonmetropolitan	2^†^		40	933,940 (9)		
**Type of visit**					39.23	<0.001
Acute	38	434,012 (39)	295	8,595,496 (81)		
Nonacute	59	672,465 (61)	75	2,023,417 (19)		

† Signifies raw visits <30 and national estimates may be unreliable.

When looking at the clinical decision-making variables of new medications, imaging and referred for visit, only the variable of referred for visit showed a significant difference between the two groups with it being higher in the neurology group (χ^2^ = 109.4; p < 0.001, Table 3). The other clinical decision-making categories of imaging and new medications prescribed did not significantly differ between the two groups (χ^2^ = 0.05 and 0.03; p = 1 and 1, respectively; Table 4).

**Table 4.** Clinical decision-making comparisons between neurology and other providers.

	Neurology only		Non-neurology		χ^2^ value	p-value
	Raw n	National estimates, n (%)	Raw n	National estimates, n (%)		
**Imaging**					0.05	1
Yes	20^†^		58	1,809,119 (17)		
No	77	902,690 (82)	312	8,809,793 (83)		
**Referred**					109.36	<0.001
Yes	67	784,760 (71)	39	1,089,559 (10)		
No	30	321,717 (29)	331	9,529,354 (90)		
**Prescribed meds**						
Yes	65	761,196 (69)	197	5,819,650 (55)		
No	32	345,282 (31)	173	4,799,263 (45)		
**New meds**	25^†^		96	2,614,060 (45)	0.03	1

† Signifies raw visits <30 and national estimates may be unreliable.

**Figure 1.**
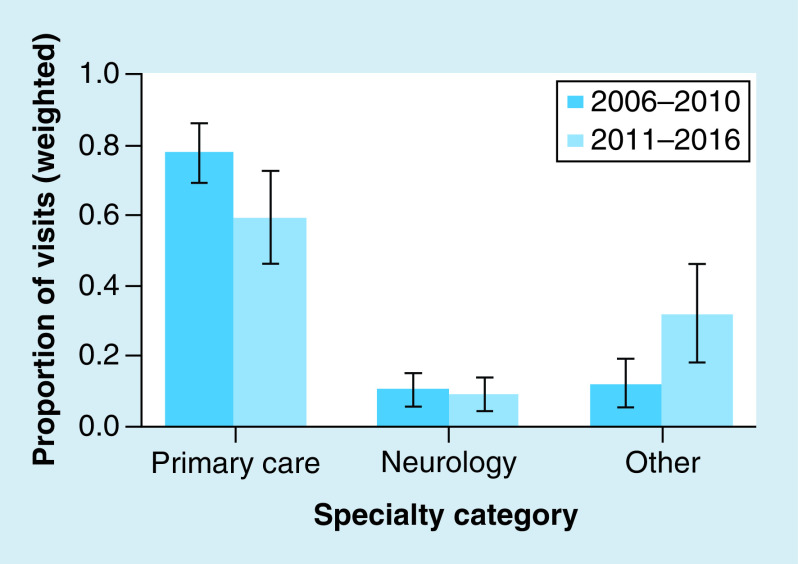
Change in provider specialty seen during study period. Proportion of visits by specialty broken down by year category (early = 2006–2010, late = 2011–2016). The error bars represent 95% CI around the estimated national proportion of visits.

The specialty seen by patients has significantly changed over time between the first 5 years of our database compared with the second 5 years (Figure 1). The ‘other’ specialty category, which is all providers who are not PCPs or neurologists, has increased with a concomitant decrease in the PCP visits while neurology visits have not changed (χ^2^ = 22.23; p = 0.0074, Figure 1). We were unable to perform formal statistical testing on the regional differences due to low sample sizes in the dataset.

## Discussion

Concussions are a common injury, especially in sports, with initial estimates of 800,000 outpatient visits per year and newer estimates of over 1 million outpatient concussion visits every year [1,2]. Our study examined the demographics and clinical decisions of concussion visits among the different specialties that provide ambulatory concussion care with a focus on comparing neurologists to the other concussion providers. We found that neurologists take care of older individuals and are involved later in the care of concussed patients. In addition, we found no significant differences in terms of clinical decision making such as obtaining imaging or frequency of prescribing new medications.

Using the nationally representative NAMCS dataset, we found that most concussion visits are performed by PCPs with over 7 million visits performed during the 10-year study period. Of note, our study showed neurologists are involved in adults’ care – patients older than 18 years old – significantly more than other specialties. In addition, we showed neurologists are more often involved in the later-stage care of concussions. This had not been described in previous studies as few studies have looked at the specialty breakdown with no known studies directly comparing neurologists with other concussion providers. A neurologist being less involved in acute care is not necessarily surprising based on the recovery timeline of most concussions with most recovering in 2–4 weeks [4,5,17]. As most concussions recover with symptomatic management and rest, there is often little need for further specialty care. The care provided by neurologists specifically and how it compared with other specialty providers was another focus.

Looking at several clinical decision-making variables helped provide insight into how different specialties managed concussions. We found that only the number of patients referred for the concussion visit was much higher in the neurology group than other providers; this is consistent as the large numbers of patients were seen by a PCP, which does not require a referral. The frequency of imaging and prescription of new medications by neurologists was overall not different from other physicians. This lack of difference initially suggests that neurologists may not be doing much different than what has already been done for the patient. However, it may be that neurologists perform such detailed neurologic assessments that serve as advanced diagnostic testing, making further testing or treatment unnecessary. Also, some proportion of patients may be referred to assess whether symptoms are attributable to concussion or due to other neurologic processes. The classes of new medications prescribed could not be directly compared between the groups due to low frequencies, which could prove useful in further describing differences as neurologists may be prescribing more nuanced and potent symptom management medications. The more nuanced or even general medications for symptom management are not described in the AAN’s position statement for SRC management [6]. The AAN has a strong focus on SRC in their position statement, which may not accurately reflect a neurologists’ panel of concussion patients.

In addition to the general management decisions among providers, we examined trends of visits during the study period. The trends over the study period showed that physicians other than PCPs and neurologists have had an increasing proportion of concussion visits. Our ‘other’ category did include a variety of specialties including medical and surgical subspecialties. This increased trend in the other providers could possibly be a result of increasing sports medicine specialists/clinics involved in concussion care, as well as the development of specialized concussion clinics. In addition, the new understanding that multidisciplinary care is important in the care of concussed patients could also help explain this shift. This shift in multidisciplinary care likely is a result of concussions producing symptoms in multiple domains, such as dizziness, difficulty focusing, blurry vision and loss of energy, which are often addressed through symptomatic management with a variety of medication classes [18]. In addition to medications, there have been small studies showing multidisciplinary rehabilitation therapies, vestibular and oculomotor rehabilitation, physical therapy and the protocolized return to activity can help with concussion recovery [19]. The multimodal and multispecialty approach to concussion has become common and may help explain the trend described above.

Using the NAMCS database has several limitations which relate to our study. The NAMCS is designed to provide national estimates; however, some may be inaccurate when the visit number is less than 30, which is seen in Table 1. In addition, the lack of imaging localization information in the database is a limitation as the concussion injury may not have been the reason for imaging but rather needed for a concomitant injury. The mechanism of injury can also be hard to determine as the mechanism can be determined only by billing codes, which can sometimes be vague or nonspecific.

The next opportunities for research would be to assess clinical decision-making differences among the specialties by looking at data that are more granular specifically the types of new medications prescribed, location of imaging and referrals made at the visit. In addition, utilizing e-codes could prove helpful in delineating the mechanism of injury, which is available in NAMCS database, but only since 2014 and could not provide enough visits for statistical testing.

## Conclusion

Our specific focus on neurologists showed that they are involved in the concussion care of older individuals and more subacute concussion management. Neurologists prescribe new medications and perform imaging in similar frequency to other concussion providers. Non-neurology and non-PCP providers have become more involved in concussion care over our study period.

## Future perspective

Concussions are a common injury with various symptoms and presentation after the injury. The multidimensional symptoms have led to medication use for symptom management; however, better understanding of subacute concussion pathophysiology will help provide more targeted therapies and rehab modalities. In addition, the multidimensional nature of concussion will continue to push multispecialty care for these patients whether directed through a single provider or through a multidisciplinary clinic.

**Summary points**

- Many specialties are involved in concussion care due to concussion’s various symptoms.
- Neurologists tend to see older patients and get involved later in concussion care.
- There are no significant differences among the providers in terms of rate of new medications prescribed and imaging performed.
- Non-neurologist subspecialties have become more involved in concussion care over time.
