# Corrigendum

**DOI:** 10.2217/cnc-2020-0008c1

**Published:** 2020-11-09

The research article ‘Demographics and management of outpatient concussion visits among neurologists and non-neurologists: 2006–2016’ by Patrick D Asselin & R Mannix, which appeared in the September 2020 issue of *Concussion* 5(3), CNC79, was published with the incorrect first figure on PubMed. This has now been corrected and the right figure is available. In addition, the article was first published with author's first name cited on PubMed as ‘PD Asselin’. This has now been corrected to ‘Patrick D Asselin’.

The correct figure is shown below:


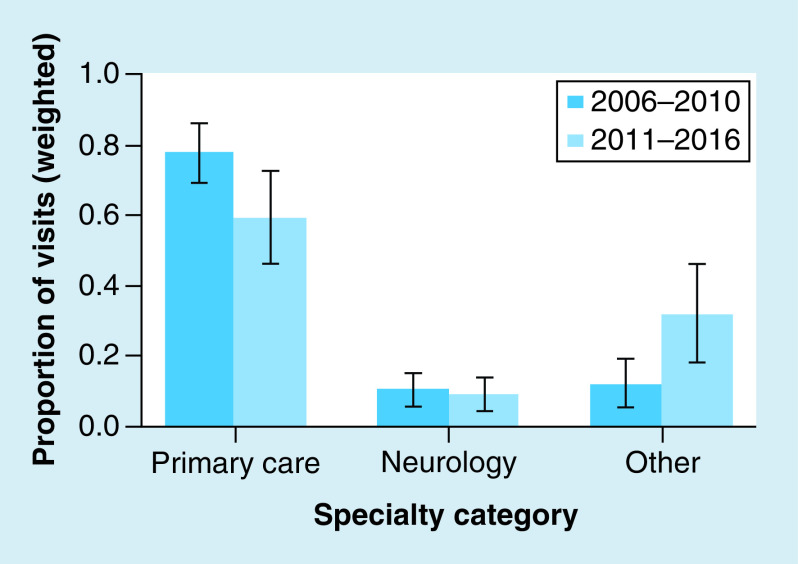


The authors and editors of *Concussion* would like to sincerely apologize for any inconvenience or confusion this may have caused our readers.

