# Corrigendum

**DOI:** 10.2217/cnc-2020-0008c2

**Published:** 2021-04-09

The research article ‘Demographics and management of outpatient concussion visits among neurologists and non-neurologists: 2006–2016’ by Patrick D Asselin and Rebekah Mannix, which appeared in the September 2020 issue of *Concussion* 5(3), CNC79, was first published with author's first name cited on PubMed as ‘R Mannix’. This has now been corrected to ‘Rebekah Mannix’.

The authors and editors of *Concussion* would like to sincerely apologize for any inconvenience or confusion this may have caused our readers.

